# Preparing for Medical Internship: A Case-Based Strategy to Teach Management of Common Overnight Calls to Students

**DOI:** 10.15766/mep_2374-8265.10966

**Published:** 2020-09-23

**Authors:** Erica Lescinskas, Zaven Sargsyan, Uma S. Ayyala, Joslyn Fisher

**Affiliations:** 1 Associate Professor, Department of Medicine, Baylor College of Medicine; 2 Assistant Professor, Department of Medicine, Baylor College of Medicine; Michael E. Debakey Veterans Affairs Medical Center

**Keywords:** Cased-Based Learning, Simulation, Internship Preparation, Psychiatry, Transition Course, Cross-cover, Virtual Learning

## Abstract

**Introduction:**

More medical schools are offering a transition-to-intern-year course to better prepare graduates for residency. Sessions where students simulate receiving cross-cover calls are frequently included and highly rated. However, simulated sessions are often resource intensive and therefore challenging to implement in all schools. We developed a case-based exercise to address this need.

**Methods:**

In 2009, our school implemented a required course focused on the transition-to-intern year, including a common overnight calls (COC) module. Students rotated through different stations in small groups which were each led by a facilitator. Topics have evolved in response to feedback, and current topics included altered mental status, chest pain, and other frequent calls.

**Results:**

Over 1,000 students have participated in the module. The students consistently reported that they perceived themselves to be more prepared for internship. Between 2009 and 2016, the mean rating of “the COC module helped prepare me for internship” was 6.29 on a 7-point Likert scale (1 = *strongly disagree* and 7 = *strongly agree*). The 2017 data are limited. In 2018 and 2019, students continued to feel more prepared for their intern year, 4.72 in 2018 and 4.71 in 2019 on a 5-point Likert scale (1 = *strongly disagree* and 5 = *strongly agree*). The students perceived the COC format as effective.

**Discussion:**

Small-group case-based classroom simulations are an effective way to improve students’ perceived preparedness for responding to common overnight calls during intern year.

## Educational Objectives

By the end of this activity, learners (graduating medical students) will be able to:

1. List common causes of altered mental status and chest pain in a hospitalized adult.
2. Initiate evaluation for altered mental status and chest pain in a hospitalized adult.
3. Demonstrate ability to manage common patient-related issues that arise overnight in the hospital.
4. Recognize when to ask for help while cross-covering patients overnight.

## Introduction

Over the last 15 years, recognition of the need to reform undergraduate medical education and particularly to enhance the fourth-year experience have led to the burgeoning of capstone courses across the US.^1–3^ Studies have shown that residency program directors want fourth-year students to better apply knowledge to patient care and, as per the ACGME core competency, to develop *advanced clinical reasoning*.^4^ Medical students find value in flexibility during their fourth year; however, they also describe the purpose of this year as a time to acquire clinical skills and to address emotional issues such as fear of independent patient care.^5^

As of 2015, 59% of medical schools required a fourth-year capstone or intern preparatory courses to ease the transition to residency.^6,7^ While the duration and content of capstone courses vary by institution, answering common calls from nurses is one of the most frequently included, and best-received sessions.^8–10^ Medical schools have shown positive outcomes and effective use of high-fidelity simulation and/or numerous personnel to teach about common cross-coverage calls, either during a subinternship or a capstone course.^10–16^ However, the need for significant resources (such as special equipment and committed personnel) may limit this practice at many schools. Fortunately, some studies have shown that technology-unenhanced, or case-based learning, is also an effective training method for clinical decision making.^17,18^ Though the literature on the fourth-year curricula has expanded, Heidemann and coauthors recently found the persistent paucity of coursework teaching students how to manage overnight calls.^7^

In 2009, as part of our institution's curricular reform, under the direction of the curriculum committee and its fourth-year task force, the newly formed APEX:MS2MD capstone course incorporated a module on common overnight calls (COC). This module, designed as a small-group, case-based, table-top exercise held in a low-stakes, supportive learning environment, has been successfully sustained over 10 years with consistent positive student feedback and students reporting greater perceived preparedness for internship. The module's relatively simple structure has allowed the flexibility to evolve to changing curricular needs without losing the module's integrity. Curricular innovations such as those found in *MedEdPORTAL* for addressing cross-coverage during internship exist; however, some of these materials required the use of high numbers of faculty and/or specific simulation equipment that may not be readily available at all institutions.^10,11^ We described the process and procedure for implementation of a low resource-requiring and adaptable COC module.

## Methods

### Curriculum Development

Our institution has required a capstone course (APEX:MS2MD) for graduating medical students since 2009. The 2-week capstone course has included a 2.5-hour mandatory interactive small-group module on how to approach COC for hospitalized patients. To guide curricular refinement in the first few years of the course, students were invited to respond to a written precourse assessment that included the question, “What are your concerns about starting internship?” Examples of common responses include:

• “I feel like I have forgotten a lot and don't know a lot of practical knowledge to answer pages and questions.”
• “First night of being on call.”
• “Having the ability to respond to inpatient emergency situations and create adequate differentials.”
• “I am especially concerned about night shifts and what to do while cross-covering patients that I do not know as well.”

The topics covered in the COC module have evolved in response to feedback and changing curricular needs. The topics have consistently included altered mental status and chest pain. In 2017, a session on other frequent calls (such as hyperglycemia and fever) was added. Over the last few years, separate COC options have been added for students pursuing surgical, obstetric, and pediatric internships. The materials and evaluations herein focus on the COC module for the adult medical inpatient.

During the 2.5-hour session, students in small groups rotated through three classroom stations, each covering a different topic. In the latest iteration of the module (used in 2018 and 2019 and materials presented in this publication), the stations were: (1) chest pain, (2) altered mental status, and (3) other frequent calls. Students participated in facilitated case-based discussions where they solved problems by gathering relevant information in a stepwise manner, reviewing available studies (e.g., labs, images, EKGs), and determining specific action steps.

Cases for each topic were developed in an iterative process with the input of generalist and specialist faculty (including geriatrics, cardiology, pulmonary, neurology, radiology, and neurosurgery) as well as residents. Relevant studies such as EKGs, radiologic images, and lab reports were collected to accompany the cases when needed. Based on student feedback, a handout with tips for managing frequent overnight calls was created and was distributed at the end of the session.

### Materials Required

The minimum number of required classrooms (or separate stations) was three–one for each topic: chest pain, altered mental status, and other frequent calls. The stations or classrooms accommodated eight to 10 students and one facilitator. We found that the stations should be separated into individual classrooms where possible to minimize distractions from competing stations (topics). Each small-group room (station) included the following materials in a folder:

• 1 facilitator guide with answers (Appendix A)
• 30 student case handouts (Appendix B)
• 5-10 copies of relevant images (ECG and head CT) (Appendix C)
• 30 copies of the tips for managing frequent calls handout–distributed upon conclusion of the rotation through the other frequent calls station (Appendix D)
• 10 student evaluation forms, completed after the third/final station (Appendix E)

Each room had dry-erase markers and a dry-erase board. Times for the group to rotate, and directions on which rooms to go to next, were clearly written on the board.

### Small-Group Facilitators

Academic clinicians, chief residents, and senior internal medicine residents served as small-group facilitators. Each facilitator was assigned to one of the three topics (chest pain, altered mental status, or other frequent calls) based on their personal preference. In advance of the course, the facilitators were emailed materials that included: (1) facilitator guide with tips for small-group facilitation (Appendix A), (2) student cases (Appendix B), and (3) relevant supplemental material/images (Appendix C). Upon arrival to their assigned COC room, the facilitators remained in their room (station) while the medical students rotated through the three different topic rooms (stations). Facilitators encouraged each student to take turns reading the cases aloud. Students would then volunteer to respond to written prompts. The facilitator guide included tips for engaging all learners.

A facilitator to student ratio of 1:8 to 1:10 was both effective and practical in our course. The total number of faculty needed depended on the total number of learners, as well as whether the module was offered more than once. In our case, we offered the module one time each year. Essentially, a minimum of three small-group facilitators were needed for each group of 30 students. For example, if 120 students were to complete the module at one setting, 12 facilitators are required, organized into four pods, with each pod including three stations (or separate rooms). Alternately, if students are divided into two groups of 60 to complete the module in two settings, then only six small-group facilitators are needed at each setting.

### Learners

All graduating medical students selected which COC module in which to participate. For years 2009 to 2016, students had two options: adult (medical) or pediatric common overnight calls. From years 2017 through 2019, we added two additional options: obstetrics/gynecology and surgery. Therefore, the number of students participating in our module was lower than in previous years. Each student participating in the adult COC module was assigned a starting room (station). Students were directed to their assigned starting room via email notice and in the first room (station) of the rotation the facilitator provided a brief overview of the format. Upon arrival to their assigned room, student case handouts (Appendix B) were distributed. Students were asked questions incrementally by the facilitator. Learning points were highlighted both during the case discussion and reemphasized upon conclusion of the 40-minute session.

Each small group of eight to 10 students rotated through the three topic rooms (chest pain, altered mental status, other frequent calls) based on a preassigned order. Each topic discussion was assigned 40 minutes, with a 5-minute break allotted for rotation to the next room (or station). A diagram of the process is provided in the Figure.

**Figure.**
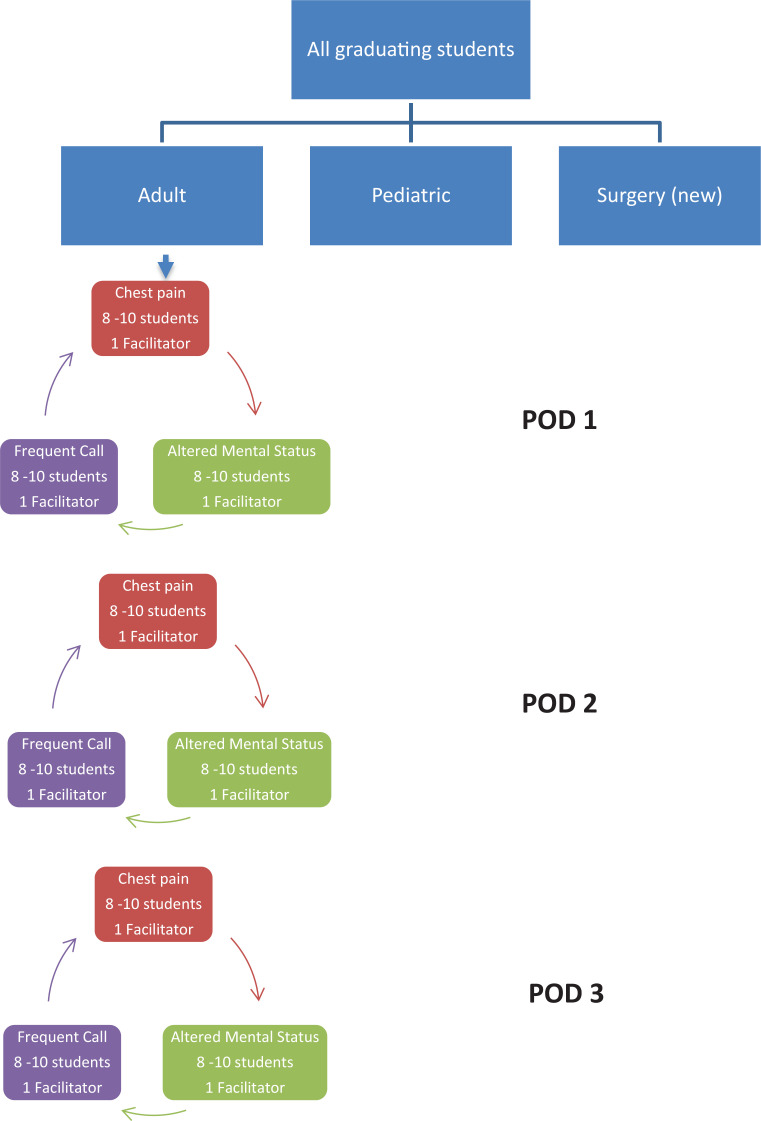
Organization of common overnight calls module.

### Evaluation

Each year the participating students completed an evaluation of the capstone course. Students had the opportunity to provide quantitative and qualitative feedback on the specific course modules including COC. Specific annual course evaluation format and process have varied slightly based on the institution's standard evaluation process at the time. Evaluations have included electronic (e-value) and paper format. In 2018 and 2019, students were also asked to complete a brief paper evaluation of each COC topic immediately upon completion of the entire COC module.

## Results

Between 2009 and 2019, over 1,000 students participated in the COC module. The evaluation format has undergone multiple changes over the past 10 years. We presented relevant results from prior course iterations; however, we focused on the results of the two most recent years, 2018 and 2019.

Early in the capstone course development (years 2009 and 2010), students completed pre- and postcourse assessments based on the institution's core competency graduation goals (CCGGs). The graduating medical students had a statistically significant improvement in their perceived self-competency between pre- and postcourse assessments for the two CCGGs most applicable to the COC module: “Indicators of medical emergencies and steps of initial evaluation and care,” and “Apply verbal and written medical communication skills to basic medical scenarios.” In addition, for the years 2009 to 2016, with an average of 137 student respondents per year, the COC module received a mean rating of 6.29 (7-point Likert scale where 1 = *strongly disagree* to 7 = *strongly agree*), in response to the statement, “I feel this session helped prepare me for internship.”

Evaluations of the latest iteration of the course delivered in 2018 and 2019 were summarized in Table 1. Due to changes in course structure with more subspecialty COC offerings, fewer total students participated in 2018 and 2019. Combined evaluation response rate was 76% (131 of 173 participants) for the adult medical COC module. Students reported that all three topics helped prepare them for intern year, and rated chest pain the highest both years. The students found the format to be effective. The tips for managing frequent calls handout, added in 2019 in response to prior comments, received positive verbal and written feedback.

**Table 1.**
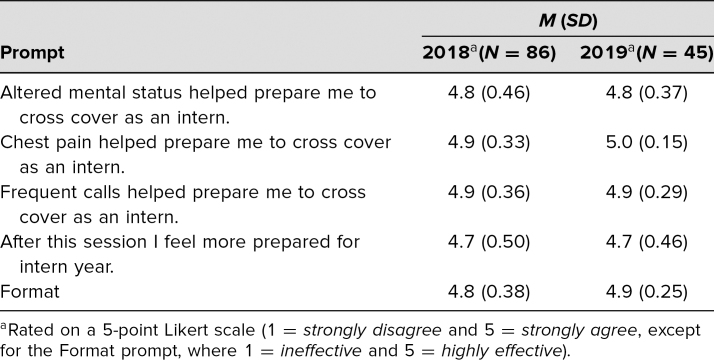
Common Overnight Calls Module Evaluations in 2018 and 2019

Since 2009, the COC module has been consistently rated in the top 10% of all offerings during the capstone course. Common themes that emerged from the authors’ review of postcourse evaluation comments over the years included practical material, how they learned to address urgent medical issues, greater comfort and confidence for starting internship, and an interactive and engaged faculty. Representative comments about the COC module are listed in Table 2.

**Table 2.**
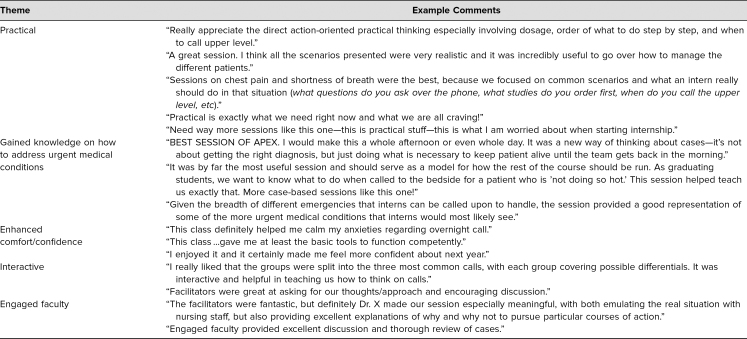
Evaluation Themes from Student Feedback

## Discussion

Capstone courses provide the opportunity to address real and perceived gaps for graduating medical students transitioning to internship. The COC module demonstrated that a case-based strategy is an effective way to teach fourth-year medical students how to approach common scenarios they will likely encounter as interns cross-covering overnight. Students rated each of the topics highly, and the majority of the students believed they were more prepared for intern year after the session. The students also rated the format as effective. The content of the COC module addressed key cross-cover challenges as described in recent literature including the management of life-threatening issues, interweaving escalation decisions, and knowing when to call for help.^7^

The strengths of the COC module included its sustainability, flexibility, and economic feasibility. The session has been successfully implemented in our institution for over 10 years. Though the cases have evolved over time, content has been rated as highly relevant for preparing for the intern year. Iterative program assessments and modifications have improved the quality of the educational product's latest version. The module's success has prompted the creation of similar modules for students pursuing surgical, pediatric, and obstetrics-gynecology internships, allowing for even smaller group sizes for the adult module. Few resources (space, facilitators, technology) are needed to operationalize this course.

As noted from the evaluations, facilitator selection was another strength of the session and was critical for the success of the curriculum. Students frequently commented that the facilitators gave outstanding pearls, and they noted that residents and chief residents were effective small-group leaders. Facilitators did not require specific faculty development for this session, beyond the written facilitator guides. Some students also commented that the rotating format kept them more engaged for a long session.

This curricular design demonstrated positive outcomes, yet several limitations exist. First, it has only been implemented at a single institution, though it was modeled after and shares features with capstone courses at other medical schools, thus may have the potential for replicability at diverse sites.^3,19^ With multiple small-group facilitators, some heterogeneity in student experience likely occurred. Over the years, we have addressed this challenge through more focused recruiting of facilitators and revision of the facilitator guides to include more explicit teaching points. Importantly, learner-reported session ratings and perceived preparedness do not necessarily correlate with knowledge, practice, or patient-level benefits. However, students completing the COC module do report finding value in feeling more prepared and thereby alleviating the anxiety of approaching internship.

Future directions for this module include further adaptation of cases to match consensus recommendations for cross-coverage and assessments of individual student competency and/or entrustable professional activities possibly through an OSCE format, and possibly surveying residency program directors on their assessment of performance by COC module participants.^20,21^

In conclusion, students perceived that this activity increased their preparedness for the intern year and their ability to handle common issues encountered on an intern night float rotation. With limited resources (few rooms, small number of facilitators requiring little training, low-cost materials), this model has the potential for replication at a wide variety of institutions to facilitate the transition to internship among a large number of graduating medical students.

## Appendices

Facilitator Guides.docxHandout - Student Cases.docxRelevant Images (ECGs, Head CT).docxHandout - Student Tips.docxStudent Evaluation of Module.docx
All appendices are peer reviewed as integral parts of the Original Publication.

## References

[1] Walling A, Merando A The fourth year of medical education: a literature review. Acad Med. 2010;85(11):1698–1704. 10.1097/acm.0b013e3181f52dc6 20881826

[2] Stevens CD Commentary: taking back year 4: a call to action. Acad Med. 2010;85(11):1663–1664. 10.1097/acm.0b013e3181f53487 20980849

[3] Teo AR, Harleman E, O'Sullivan PS, Maa J The key role of a transition course in preparing medical students for internship. Acad Med. 2011;86(7):860–865. 10.1097/acm.0b013e31821d6ae2 21617513 PMC3128667

[4] Lyss-Lerman P, Teherani A, Aagaard E, Loeser H, Cooke M, Harper GM What training is needed in the fourth year of medical school? Views of residency program directors. Acad Med. 2009;84(7):823–829. 10.1097/acm.0b013e3181a82426 19550170

[5] Cosgrove EM, Ryan MJ, Wenrich MD Empowering fourth-year medical students: the value of the senior year. Acad Med. 2014;89(4):533–535. 10.1097/acm.0000000000000191 24556780 PMC4885582

[6] Elnicki DM, Gallagher S, Willett L, et al Course offerings in the fourth year of medical school: how US medical schools are preparing students for internship. Acad Med. 2015;90(10):1324–1330. 10.1097/acm.0000000000000796 27002885

[7] Heidemann LA, Fitzgerald JT, Hartley S Are medical students trained in cross-cover? Clin Teach. 2019;16(3):214–219. 10.1111/tct.12803 29947072

[8] Esterl RM Jr., Henzi DL, Cohn SM Senior medical student “boot camp”: can result in increased self-confidence before starting surgery internships. Curr Surg. 2006;63(4):264–268. 16843778 10.1016/j.cursur.2006.03.004

[9] Fisher JW, Thompson BM, Garcia AD Integrative clinical experience: an innovative program to prepare for internship. Teach Learn Med. 2007;19(3):302–307. 10.1080/10401330701366788 17594227

[10] Tischendorf J, O'Connor C, Alvarez M, Johnson S Mock paging and consult curriculum to prepare fourth-year medical students for medical internship. MedEdPORTAL. 2018;14:10708 10.15766/mep_2374-8265.10708 30800908 PMC6342441

[11] Chakraborti CA A simulation-based curriculum for 4th-year medical students during an internal medicine acting internship. MedEdPORTAL. 2009;5:1687 10.15766/mep_2374-8265.1687

[12] Fredrick NB Acting intern on call simulations. MedEdPORTAL. 2012;8:9076 10.15766/mep_2374-8265.9076

[13] Wald D, Peet A, Cripe J, Kinloch M A simulated night on call experience for graduating medical students. MedEdPORTAL. 2016;12:10483 10.15766/mep_2374-8265.10483 30984825 PMC6440402

[14] Frischknecht AC, Boehler ML, Schwind CJ, et al How prepared are your interns to take calls? Results of a multi-institutional study of simulated pages to prepare medical students for surgery internship. Am J Surg. 2014;208(2):307–315. 10.1016/j.amjsurg.2014.01.014 24933670

[15] Healey A, Sherbino J, Fan J, Mensour M, Upadhye S, Wasi P A low-fidelity simulation curriculum addresses needs identified by faculty and improves the comfort level of senior internal medicine resident physicians with in hospital resuscitation. Crit Care Med. 2010;38(9):1899–1903. 10.1097/ccm.0b013e3181eb3ca9 20639751

[16] Laack TA, Newman JS, Goyal DG, Torsher LC A 1-week simulated internship course helps prepare medical students for transition to residency. Simul Healthc. 2010;5(3):127–132. 10.1097/sih.0b013e3181cd0679 20651473

[17] Norman G, Dore K, Grierson L The minimal relationship between simulation fidelity and transfer of learning. Med Educ. 2012;46(7):636–647. 10.1111/j.1365-2923.2012.04243.x 22616789

[18] Schwind CJ, Boehler ML, Markwell SJ, Williams RG, Brenner MJ Use of simulated pages to prepare medical students for internship and improve patient safety. Acad Med. 2011;86(1):77–84. 10.1097/acm.0b013e3181ff9893 21099392

[19] Clay AS, Ming DY, Knudsen NW, et al CaPOW! Using problem sets in a capstone course to improve fourth-year medical students’ confidence in self-directed learning. Acad Med. 2017;92(3):380–384. 10.1097/acm.0000000000001193 27119334

[20] Heidemann LA, Fitzgerald JT, Hughes DT, Hartley S Inpatient cross-cover consensus recommendations for medical and surgical residents: a Delphi analysis. J Grad Med Educ. 2019;11(3):277–283. 10.4300/jgme-d-18-00707.1 31210857 PMC6570444

[21] Brown DR, Warren JB, Hyderi A, et al Finding a path to entrustment in undergraduate medical education: a progress report from the AAMC Core Entrustable Professional Activities for Entering Residency Entrustment Concept Group. Acad Med. 2017;92(6):774–779. 10.1097/acm.0000000000001544 28557941

