# Supplementary material for: Preparing for Medical Internship: A Case-Based Strategy to Teach Management of Common Overnight Calls to Students
Source: MedEdPORTAL. 2020 Sep 23;16:10966. doi: 10.15766/mep_2374-8265.10966 (PMC7511063; doi:10.15766/mep_2374-8265.10966)
Supplement: Supplementary file 1 — Facilitator Guides.docxHandout - Student Cases.docxRelevant Images (ECGs, Head CT).docxHandout - Student Tips.docxStudent Evaluation of Module.docx [file mep_2374-8265.10966-s001.zip › A. Facilitator Guides.docx]

# **Facilitator Guide: Overview**

Thank you for agreeing to participate as a small group leader/facilitator for the common overnight calls module. Please review these tips prior to arriving for your session.

1. Review all material relevant to your assigned topic (Chest Pain OR Altered Mental Status OR Other Common Calls) prior to arrival. The facilitator guide provides answers to the prompts as well as additional information. There will be student copies of the cases in each room. The student copies have cases and prompts only – no answers. You should have received a copy of the student cases handout along with this facilitator guide.

2. For the first session of the module, explain to the students that you all will have 40 minutes to cover the topic. At the end of 40 minutes, the students will have 5 minutes to rotate to the next room of their assigned “pod.” The students will rotate through 3 stations in total. You will have 3 groups of 8-10 students rotate into your room.

3. Please allow each student an opportunity to speak. Students can work together as a team to figure out answers.

4. Remind the students to silence or turn off cell phones. No text messaging.

5. Remember to support a safe learning environment. What is shared in the room stays in the room. For example, participants must respect expressed feelings. Acknowledge they are very real to the person who expresses them.

7. If one case runs longer than anticipated, you might consider asking the students which remaining cases they want to cover and /or shorten the remaining cases.

8. A key element we want to address is differentiating a sick patient from those that are less acute. **Remind the students to identify if the patient is “sick” or “not sick” in each case.**

9. Another key is when to call for help. **Encourage the students to clarify in each case when they would (or should) call for help. Remind students that they are never alone.**

10. Time permitting, you may want the students to “teach back” at the conclusion of the 45-minute session and provide 1 to 3 take home points covered about the particular topic.

# **COMMON CALLS: CHEST PAIN**

### FACILITATOR COPY

Format:

- 40-minute small-group, case-based discussion. ~10 students, 1 facilitator.
- Students do not have the info in blue italics (answers) and boxes (case progression info)
- Group provided with copies of ECGs #1-4 (may need to share, and please collect at the end of session)

Suggested approach:

- Ask students to take turns slowly reading the prompts and questions
- Allow a few moments for group to process / think about questions
- Case 1 should take disproportionately more time, as it lays groundwork with respect to reviewing the major differential for chest pain and initial work up and management
- The “answers” / teaching points below are only a guide, and can be adapted as seen fit by the facilitator

**CASE # 1 (12-15 minutes):**

You are on a night float rotation and are called at 10 pm about a 52-year-old woman admitted to general surgery 2 days ago with cholecystitis, s/p cholecystectomy yesterday. She developed chest pain earlier tonight. You go to the bedside.

As you ask her to tell you about her symptoms, you should listen for suggestive descriptors or risk factors with the most concerning causes of chest pain in mind. What are your no-miss causes of chest pain?

*Answer (consider writing on board):*

- *Myocardial ischemia*
- *Pulmonary embolism*
- *Aortic dissection*
- *Pericarditis with tamponade*
- *Pneumothorax*
- *Esophageal rupture*
- *Pneumonia*

*Remember that myocardial ischemia (e.g. angina pain, ischemic ECG changes, or troponin elevation) can occur either with acute coronary syndrome/thrombosis, or from increased oxygen demand (such as from fast arrhythmia, sepsis, or severe hypertension).*

PROVIDE INFO after students respond: She has 6/10 pressure in the middle and left side of her chest. Has nausea but no SOB. Has diabetic neuropathy but no past cardiac problems or workup.

Unfortunately, the patient’s experience is not always textbook (e.g. ischemic pain in diabetics and women can be atypical). What physical exam & studies would you do in a patient with chest pain to further assess for possible diagnoses?

*Vitals are vital, and each of them might dramatically sway the differential.*

*Pulse (regularity, rate, strength)*

*JVP (elevated in tamponade, tension PTX, massive PE, or RV MI)*

*Heart sounds (e.g. arrhythmia, new murmurs or S4 indicating ischemia)*

*Lungs sounds (critical in catching a PTX or PNA + part of volume exam)*

*Chest wall (rash or tenderness)*

*Abdomen (abdominal pathology can cause chest pain)*

*Reflexive tests for CP or SOB should include:*

1. *ECG*
2. *CXR*
3. *Troponin in most patients – one now, one in 6 hours*

PROVIDE INFO: Vitals show: BP 178/100, HR 92, RR 16, Afebrile, O2 sat 95% on room air. Heart regular, a bit fast, no abnormal sounds. JVP <5cm water (normal). A few crackles at both bases, otherwise normal. Abdomen benign.

What does the EKG show? As always, focusing on rhythm and ischemia is highest-yield in acute situations. *(pass around EKG # 1 with anteroseptal STEMI)*

What are your initial management steps?

There’s a clot in her LAD: we can’t take it out. You must immediately call the STEMI pager, where the interventional cardiologist on call will hear the story from you, look at the ECG from home, and decide whether to activate the cath lab.

In the meantime, use the mnemonic MONA’S Hep B as your menu of medical therapies for any ACS (including NSTEMI and STEMI).

- Morphine: ONLY IF you can’t control the pain with nitrates and beta blockers
- Oxygen: ONLY IF hypoxemic (O2 sat < 94%!)
- Nitrates: BUT NOT IF low blood pressure or RV-MI
- Aspirin: HUGE benefit with very little down side (a no-brainer unless brain bleed)
  - - Other antiplatelets like clopidogrel – defer to cardiology
- Statin: Atorva 80 has benefit in acute setting, pretty much no downside
- Heparin: Unless high bleeding risk. LMWH or UFH ok.
- Beta-blocker: NOT IF bradycardia or low BP. Short-acting only.

What if her EKG had been completely normal, everything else the same? CXR was also normal.

*She could still be having unstable angina or an NSTEMI, but GERD & referred pain could also be very likely. An Aspirin and statin would again be a no-brainer with any suspicion. Response to sublingual nitro may be helpful diagnostically. If no help, empiric PPI, GI cocktail, or tylenol would be appropriate. Heparin would be a judgment call, but you probably don’t have enough to start it given the more marginal benefit/risk ratio. A repeat troponin 4-6 hours in addition to now would be critical for diagnosis (and rest of ACS treatment indicated if it rose). Similarly, reassessment of both chest pain and ECG evolution would be critical. Very often in this scenario the ECGs and troponins remain negative, and the pain resolves. But of course we can never assume this to begin with.*

**CASE # 2 (6-8 minutes):**

An hour later, you are called about another patient with chest pain. Mr. Loopy was admitted yesterday with altered mentation and alcohol intoxication. He had cleared up today and the team was planning discharge tomorrow. He called his nurse after a sudden onset of chest pain.

What questions would you ask the nurse immediately? Any orders?

#### Latest vitals, ask to check a new set of vitals. Ask for ECG +/- portable CXR, put in order.

*Would not ask nurse too many details of the pain – you should get the history from the patient.*

The RN is finishing up rechecking VS as you arrive: T 98, P 128, BP 135/95, RR 20, 99% RA

Mr. Loopy tells you that 10 minutes ago while watching Police Academy 4, he suddenly started having palpitations. He doesn’t quite have pain but there’s some mild pressure in his chest. He otherwise feels ok, but the racing heart is scaring him as he’s never had it before.

Any hypotheses or diagnoses to consider? What would you look for on vitals and exam? What studies would you order?

His mental status is normal and he has no tremors. JVP is flat (<5cm water). His pulse and heart sounds are irregular. Rest of exam normal.

Look at EKG # 2 and assess for rhythm and ischemia. Does it explain all of his symptoms? How would you manage this patient?

#### The patient is in symptomatic atrial fibrillation with RVR. Acutely, the main goal is control of the rapid rates.

#### BP good: metoprolol or diltiazem

#### BP low: amiodarone, or may need cardioversion if hypotensive

#### The facilitator can ask: “OK, you’ve come up with the right drug, but what dose are you going to actually order? How will you decide?” Point out that in addition to calling one’s upper level, the interns can use trusted resources like pocket-guides and Uptodate.

####

#### We have explained the patient’s symptoms, but a question remains: why is he in AF? This is important but slightly less urgent to answer. Once you believe the patient is stable, this would be another thing you could look up online, and consider further studies such as a TSH or echo.

**CASE #3 (5-7 minutes):**

You are called about a patient with acute respiratory distress and excruciating chest pain. It seemed to have started suddenly according to the nurse, but the patient is too breathless to talk. You read your signout: “24 yo M with ESRD, came in because his dialysis graft stopped functioning. Going to receive dialysis tomorrow.”

What do you think is going on?

#### Students will usually recognize uremic pericarditis, volume overload, hypertensive urgency, and PE as possibilities in the clinical context. This pattern recognition is helpful in a pinch.

What will you do now?

Vitals always first: T 97, P 120, BP 92/70, O2 85% RA, RR 40. On exam, JVs distended to >20cm water. Trachea is shifted left. There are no breath sounds on the right chest wall, which is very tympanic to percussion. Why is there a pneumothorax!? Encourage students to ask for more history (perhaps of the nurse), or to read between the lines in the sign-out on-liner. In fact, a subclavian central line was just placed in preparation for dialysis. Point out the failure of pattern recognition, especially when one is inexperienced. A more systematic approach, such as considering all life-threatening diagnoses on the board and always doing a focused physical exam, will lead to the diagnosis. Mention that the ECG simply showed sinus tachycardia and no other abnormalities. If have time can go through basics of ECG here.

How will you assess further?

The patient is critically ill. Call an RRT or code immediately. Ask someone to STAT-page SURGERY with pager and overhead. The patient needs a chest tube, and a needle decompression in the meantime (would only do this yourself if you think the patient won’t last minutes)

# **CASE #4 (5-7 min)**

A health young woman just had her first baby. She has called the nurse because she is having dizziness and palpitations.

What might be going on?

## Blood loss, pulmonary embolus, anxiety may come to mind.

## Systematically considering other life threatening diagnoses still worthwhile:

## Myocardial ischemia

- - *ACS from atherosclerosis unlikely but coronary dissection possible and more common in pregnancy/peripartum – should still look at ECG for ischemia!*
  - *Demand ischemia very possible if arrhythmia from peripartum cardiomyopathy or stress state*
- *PTX, aortic dissection, and esophageal rupture: clear risk factor may not come to mind in this lady, but extreme Valsalva during labor could cause all of these things*
- *Pneumonia or pericarditis less likely.*

## P 180 and thready. BP 70/55. RR 30, O2 94% RA. On exam she is sweaty and looks both anxious and tired.

What one test do you need at this point?

ECG. In fact, no time to wait for a full 12-lead ECG. Ask the nurse to attach a bedside Lifepak monitor.

Based on ECG # 4, what would your next few management steps be?

*REGULAR, WIDE, FAST = VENTRICULAR TACHYCARDIA. Would call a code, cause it’s about to be one. In meantime, initiate ACLS.*

*Does she have a pulse? Yes. So no CPR + EPI*

*Is she stable or unstable? Unstable. Any unstable tachycardia -> cardiovert.*

# **COMMON CALLS: ALTERED MENTAL STATUS**

### FACILITATOR COPY

Format:

- 40-minute small-group, case-based discussion. ~10 students, 1 facilitator.
- Students do not have the info in blue italics (answers) and boxes (case progression info)

Suggested approach:

- Ask students to take turns slowly reading the prompts and questions
- Allow a few moments for group to process / think about questions
- The “answers” / teaching points below are only a guide, and can be adapted as seen fit by the facilitator

**CASE # 1: (15 minutes)**

You are called about a 74 year old woman with diabetes who was admitted one day ago with pneumonia and is now confused. On your check out sheet, the patient is listed as stable and improving, with plans to go home soon.

What questions would you like to ask the nurse?

*What is her baseline, and what is happening now? (important to keep mental status up to date in the chart in progress notes)*

*What are her vitals, including oxygen saturation? If the patient has not had recent vitals, ask the nurse to obtain a new set while you are on your way to see the patient.*

*Since she is diabetic, also ask nurse to get an accucheck.*

ADDITIONAL INFORMATION: At baseline, the patient is alert and oriented to person, place, and time. She seemed at her baseline a few hours ago when nurse saw her last. Now, she is sleepy, and mumbling. The nurse just checked vitals: Afebrile, BP 135/80, HR 96, RR 16, O2 sat 97% on room air. Accucheck is pending.

As you are walking to see the patient, what is your differential for altered mental status?

*Hypoxia- O2 sat ok in this patient, quick easy test for all AMS patients*

*Hypoglycemia – given acuity of AMS, concerning, check accucheck (quick test for all AMS*

*patients, higher level of suspicion if on insulin or sulfonylurea, received insulin*

*for hyperkalemia, septic, or have liver failure)*

*Hypercalcemia- check BMP, especially concerning if history of cancer, hyperparathyroidism,*

*Hypo/hypernatremia – check BMP*

*Hypercapnic respiratory failure – check ABG, higher suspicion in patients with COPD, obesity*

*hypoventilation syndrome*

*Narcotic overdose – check medication list and when they were given*

*Infection – can be new infection*

*Intracranial pathology – stroke or bleed less likely to cause AMS unless significant bleed or edema*

ADDITIONAL INFORMATION: When you arrive at patient’s bedside, accucheck is 38.

What would you do now?

*Treat the hypoglycemia: since she is altered, give D50 by IV. If she were not altered, can treat*

*with juice.*

*Investigate why patient was hypoglycemic: Check insulin dosing, see if she is eating less than normal, and check creatinine to see if she may not be clearing insulin well.*

ADDITIONAL INFORMATION: Patient was continued on her home dose of NPH 15 units twice a day with medium dose sliding scale although her creatinine had increased to 2 from her baseline of 1.3 and she has been eating less than half her meals. Looking back, her glucoses have been 70-120 since she was admitted

*Make sure to adjust insulin dosing (likely decrease sliding scale and decrease basal) and make sure to notify team in the morning so they can continue to adjust insulin.*

**CASE# 2: (10-12 minutes)**

Mr. Smith is a 54 year old man brought to the hospital after he fell from a bridge and sustained a hip fracture. He underwent repair of the fracture, and now the nurse calls you three days into his hospital stay because he is mumbling and seeing things in the room that are not there.

What information do you need next, and what is your differential?

*What is his baseline?*

*Vitals, physical, medications, any more history?*

*Differential: pain med overdose, delirium tremens, intracranial pathology, psychiatric*

ADDITIONAL INFORMATION: T 100.3 HR 120 BP 150/100 R 22 O2 sat 99% RA. On physical, diaphoretic, tachycardic, and picking at things in the air. He is trying to get out of bed

What additional history would you like?

*How much alcohol does he drink? Has he ever had withdrawal symptoms or delirium tremens before when he tried to stop?*

ADDITIONAL INFORMATION: His mom is at bedside, and says he drinks at least a fifth of vodka a day. He has never stopped drinking before this hospitalization

What do you think is going on, and what would you do for the patient?

#### Diagnosis: Delirium tremens.

#### Treatment: thiamine, IVF, and start IV benzodiazepine since symptoms are severe. Transfer to IMU. He will need a sitter and close titration of benzodiazepines. Involve upper level early since he will need reassessment frequently and potentially ICU if does not respond to IV benzodiazepines

# **CASE #3 (10-12 minutes)**

The nurse calls you about Mrs. H, an 80 year old woman with a history of atrial fibrillation on coumadin who was admitted for chest pain. You are called overnight because she is found on the floor, confused.

# You go to see her right away. What is your differential?

#### It is unclear if she fell because she was altered, or if she became altered after fall.

#### Things that could make her altered before fall: medications, sundowning, infection, metabolic, intracranial bleed

#### Differential if altered after fall – could include intracranial bleed, although less likely to occur acutely with relatively minor trauma, and may present later

ADDITIONAL INFORMATION: She is sleepy but arousable to voice, although she keeps falling asleep and is not following commands. Her pupils are dilated and respond sluggishy to light.

Which study would you do?

#### Stat head CT without contrast

ADDITIONAL INFORMATION: Show CTs with epidural bleed, SAH, subdural hematoma and go over the differences in imaging findings for each. In this patient, she developed a subdural hematoma after a fall.

What additional steps would you take?

#### Make sure patient is protecting airway, call upperlevel and neurosurgery

When you look at the medication list you see that this patient received zolpidem 10 mg earlier that evening for sleep.

*As a physician, you will be called frequently because patients are having trouble sleeping, and patients will ask for sleep aids. If you are called overnight for a sleep aid, what should you do?*

*Obtain further history from the RN*

- - *Have they tried improving sleep hygiene (turning off the lights, decreasing sounds, turning off TV, meditation)*
  - *How long as the patient been trying to fall asleep?*
  - *Does the patient require sleep aids at home to fall asleep?*
  - *Did the patient have any caffeinated beverages this evening?*

*Review the chart*

- - *Check the home medications to see if he takes anything for insomnia*
  - *Review allergies to medications*

*Do not feel pressure to prescribe asleep aid, ideally can talk to the patient (either*

*over the phone or in person)*

- - *Highlight that Insomnia is multifactorial (anxiety, due to medical illness, poor sleep hygiene, caffeine intake)*
  - *Emphasize sleep aids have risks*

What should you suggest as a first line treatment?

*Sleep Hygiene*

- - *Dim the lights*
  - *Turn off the TV*
  - *Use earplugs or headphones if the patient would like them*
  - *Warm beverage or light snack*
  - *Reassure the patient that it can be difficult to sleep in the hospital but that this is safest option for him or her to try for at least 1-2 hours.*

*If sleep hygiene does not work or if the patient tells you he chronically takes a sleep aid then prescribe the medication he/she takes at home*

***(Caveat- if the patient takes benzos and is acutely ill you may want to avoid using this home medication)***

***Other choices:***

*Non-Hypnotic*

- - *Trazodone 50 mg (good side effect profile, genuinely effective, can cause daytime sleepiness, caution with heart disease can prolong QTc)*

*OTC*

- - *Diphenhydramine 25 mg (can have paradoxical reaction, not very effective, can cause daytime drowsiness, avoid in elderly or those with dementia if possible)*
  - *Melatonin 0.5-1 mg*

*Hypnotics (would not start inpatient especially on someone with medical problems, may be ok to give if patient has been taking chronically and is stable*

- - *Zolpidem (Ambien) 5 mg, Lunesta, Sonata*

*It is OK to defer to the primary team the following day. Sleep Aids should not be*

*given after midnight as it will just cause daytime sleepiness.*

*****If you have extra time, can discuss sundowning, and how to recognize it in our patients.*

# **COMMON CALLS: Frequent Calls**

### FACILITATOR COPY

Format:

- 40-minute small-group, case-based discussion. ~10 students, 1 facilitator.
- Students do not have the info in blue italics (answers) and boxes (case progression info)

Suggested approach:

- Ask students to take turns slowly reading the prompts and questions
- Allow a few moments for group to process / think about questions
- The “answers” / teaching points below are only a guide, and can be adapted as seen fit by the facilitator

**Fever**

**Case (8-10 minutes)**

*You are called overnight about a 44 year-old woman with leukemia who has been in the hospital for 2 weeks for induction chemotherapy, who has a temperature of 101 degrees Fahrenheit.*

1. **What information do you need?**

*Ask the nurse for a complete set of vitals.*

*Look on the sign out sheet and chart for other information (in this case, neutropenia)*

ADDITIONAL INFORMATION: Temp 101, HR 88, BP 110/70 (same as baseline), RR 14 , O2 sat 99% on room air. When you look in the chart, you note that her absolute neutrophil count that morning was 100

1. **What should you do next?**

*Evaluate the patient for symptoms such as cough, dysuria, tenderness at a line site that could point to source of infection.*

*Since this patient has neutropenic fever, draw blood cultures and start an antibiotic that*

*has coverage against pseudomonas (such as cefepime or meropenem). May also need*

*Vancomycin*

1. **What if the patient did not have leukemia and was admitted with chest pain?**

*No evidence of sirs – evaluate for source (including symptoms/signs such as cough, sore*

*throat, cold/flu symptoms, dysuria, abdominal pain/diarrhea, skin for soft tissue infections or ulcers, and in an inpatient that includes IV sites/old sites for signs thrombophlebitis).*

*If patient has an indwelling venous access device or suspicion of blood stream infection would order blood cultures. Utility in average inpatient is low.*

*If patient is stable and no source, can observe, and does not necessarily need empiric*

*antibiotics (if patient appears sick, would give empiric antibiotics).*

*If patient is uncomfortable can give acetaminophen, but no need to treat just based on*

*temp.*

**Common Calls Facilitator Guide**

**Hypertension**

**Case 1 (5-8 minutes)**

*A 53-year-old female with end stage renal disease on intermittent emergent hemodialysis was admitted earlier in the day for signs of volume overload, typical of pre dialysis symptoms. She received one session of hemodialysis (removal of 2L) with plan for another session of dialysis in the morning. Overnight, the nurse calls you for elevated blood pressure to 168/90.*

**What would you ask the nurse?**

- *Obtain further history*
  - *What is the patient doing? Is the patient symptomatic?*
  - *Specific symptoms to elicit*
    - *Headache*
    - *Vision Disturbance*
    - *Chest Pain*
    - *Shortness of Breath*
    - *Pain*
- *Ask for a full set of vitals*

ADDITIONAL INFORMATION: The nurse tells you that the full set of vitals are temp 98.6, BP 168/90, HR 84, RR 12, SpO2 96% on room air. The patient is resting in bed watching television and has no specific complaints.

**What should you do next?**

- *Review the chart*
  - *Look at the blood pressure trends since admission*
  - *What is the patient’s current medication regimen?*
  - *When did she receive her meds? Review the medication administration record. Were any doses held? When is the next dose due?*
- *If there are any concerns, err on the side of seeing the patient to evaluate for symptoms, and if something else (like pain, anxiety, agitation) could be causing the elevated blood pressure, before you consider giving a medication*

ADDITIONAL INFORMATION: On review of the chart, you note that the patient has had elevated blood pressure since admission ranging from 150-180/80-100. She is on nifedipine 90mg and her last dose was at 9AM in the morning.

**How should you treat the patient’s blood pressure?**

*No treatment*

*Not all elevated blood pressure inpatient needs immediate treatment. Clues to recommending no treatment include that the patient is (1) asymptomatic (2) within her blood pressure range (3) has ESRD on HD and likely has elevated blood pressure at baseline. The patient is planned to undergo HD in the AM which will likely also improve her blood pressure. It is more important to treat the patient rather than the number. Although she is above her blood pressure goal, controlling it is a long term issue, and there are risks with using drugs to acutely lower blood pressure.*

**Case 2 (5-8 minutes)**

*A 48-yo-male with HTN ran out of all his home meds about 2 weeks prior and presented to the EC earlier in the day with complaints of chest discomfort. His blood pressure at the time was 220/110. EKG did not show any signs of ischemic changes. He was given medication which improved his blood pressure and the chest discomfort resolved. Overnight, the nurse calls you to let you know that the patient’s blood pressure is now 190/100.*

**What would you ask the nurse?**

- *Obtain further history*
  - *What is the patient doing? Is the patient symptomatic?*
  - *Specific symptoms to elicit*
    - *Headache*
    - *Vision Disturbance*
    - *Chest Pain*
    - *Shortness of Breath*
    - *Pain*
- *Ask for a full set of vitals*

ADDITIONAL INFORMATION: The nurse tells you that the full set of vitals are temp 98.6, BP 194/100, HR 82, RR 14, SpO2 98% on room air. The patient is complaining of chest discomfort similar to what he had at time of presentation.

**What should you do next?**

- *Go see the patient*
- *Review chart to see what medications were given and when*
- *Low threshold to call your upper level*

*This patient’s presentation is very different from the last case. Any patient with chest pain needs to be evaluated in person quickly.*

ADDITIONAL INFORMATION: You assess the patient with your upper level. An EKG is unchanged. You suspect that the patient’s symptoms are because of his elevated blood pressure. On review of the chart you note that the patient initially received labetalol 20mg IV on admission which decreased his blood pressure to 178/80. No further meds were given.

**How should you treat this patient?**

- *Medication choice depends on the severity of the patient’s symptoms. Generally, PO is preferred over IV medications in non-emergent situations.*
- *If the patient is having true hypertensive emergency, then IV medications can be considered. IV labetalol is a good choice if the patient’s HR can tolerate. For emergencies, IV drips such as nicardipine may be necessary since you can titrate them and avoid lowering blood pressure abruptly. Since the patient responded well to IV labetalol before, you could try another dose now. Always be cautious to avoid lowering blood pressure too quickly.*
- *Consider restarting patient’s home medications slowly now. Adding oral agents will help continue to bring down the patient’s blood pressure and avoid another situation such as this one in the future after the IV medications wear off.*
- *The goal lowering of BP should be about 25% in first 24 hours. Dropping blood pressure too quickly can cause ischemic symptoms.*

**Common Calls Facilitator Guide**

**Pain**

*Case (5 minutes)*

*A 33 year-old female is admitted for an asthma exacerbation. She has improved throughout her hospital course and the plan is for discharge tomorrow. The nurse calls you to report the patient is suffering from a headache and is requesting an order for a pain medication.*

What do you do next?

- - *Ask for vital signs*
  - *Ask if this headache a new symptom?*
  - *Are there alarm features (photophobia, nausea/vomiting, visual disturbance, neurologic symptoms)?*

ADDITIONAL INFORMATION: After reviewing the chart and speaking with the patient you learn that she does have a headache about once a month for the past several years. This headache is similar to her prior headache, and she does not have any other symptoms. Vital signs and physical exam are normal. She usually takes Tylenol or advil at home which relieves the headache.

***What medications (if any) would you recommend?***

- - *In this case, since it is not a new symptom, she does not need further work up*
  - *Oral acetaminophen 500mg – 650mg would be appropriate for mild pain*

**Hyperglycemia**

***Case (10 minutes)***

*At 9 pm, the nurse calls you about a 54 year old man with diabetes with a glucose of 360. From your check out sheet, you see that he was admitted today with chest pain, cardiac enzymes are negative, and a stress test is planned tomorrow.*

What do you do next?

*Ask the nurse when the patient last ate (and what he ate); if he just ate a piece of cake that the family brought him, it is a different situation than if he last ate his dinner tray 4 hours ago.*

*Also ask if he is having nausea, vomiting or other symptoms.*

*Look in the chart to see what his glucoses have been. Check diet to make sure it is diabetic, see what medications he was taking at home and if he has received any insulin since he has been here. Also check to make sure he is not on dextrose containing fluids. Look at BMP to make sure there was no acidosis or evidence of a gap earlier. If having nausea and vomiting, would consider repeating BMP*

ADDITIONAL INFORMATION: After reviewing the chart and speaking with the patient you learn that he takes metformin at home, and that he last ate a diabetic dinner 3 hours ago. His glucose before dinner was 200, and by the sliding scale he received 2 units of insulin at that time. He does not have any nausea or vomiting now, and the bicarb was normal on the admission BMP a few hours ago. He is not on any medication other than sliding scale for diabetes as an inpatient, and he has an order to be npo after midnight for stress test tomorrow

How would you manage this?

*He will likely receive some short acting insulin by nighttime sliding scale (less coverage than he would receive for the same glucose premeal). Since he is npo, and only on metformin at home, could just give that coverage and recheck in AM. In general, there is data to support placing inpatients on long acting insulin when we take them off their oral meds, but bedtime when the patient is npo may not be the best time to start this.*

*Would also try to discuss long acting and short acting insulin with students.*

****If you have extra time, can discuss basic prns for nausea, and constipation*
