# Supplementary material for: Preparing for Medical Internship: A Case-Based Strategy to Teach Management of Common Overnight Calls to Students
Source: MedEdPORTAL. 2020 Sep 23;16:10966. doi: 10.15766/mep_2374-8265.10966 (PMC7511063; doi:10.15766/mep_2374-8265.10966)
Supplement: Supplementary file 1 — Facilitator Guides.docxHandout - Student Cases.docxRelevant Images (ECGs, Head CT).docxHandout - Student Tips.docxStudent Evaluation of Module.docx [file mep_2374-8265.10966-s001.zip › B. Handout - Student Cases.docx]

# **COMMON CALLS: CHEST PAIN**

### STUDENT COPY

**CASE # 1**

You are on a night float rotation and are called at 10 pm about a 52-year-old woman admitted to general surgery 2 days ago with cholecystitis, s/p cholecystectomy yesterday. She developed chest pain earlier tonight. You go to the bedside.

As you ask her to tell you about her symptoms, you should listen for suggestive descriptors or risk factors with the most concerning causes of chest pain in mind. What are your no-miss causes of chest pain?

Unfortunately, the patient’s experience is not always textbook (e.g. ischemic pain in diabetics and women can be atypical). What physical exam & studies would you do in a patient with chest pain to further assess for possible diagnoses?

What does the EKG show? As always, focusing on rhythm and ischemia is highest-yield in acute situations.

What are your initial management steps?

What if her EKG had been completely normal, everything else the same? CXR was also normal.

**CASE # 2**

An hour later, you are called about another patient with chest pain. Mr. Loopy was admitted yesterday with altered mentation and alcohol intoxication. He had cleared up today and the team was planning discharge tomorrow. He called his nurse after a sudden onset of chest pain.

What questions would you ask the nurse immediately? Any orders?

Look at EKG # 2 and assess for rhythm and ischemia. Does it explain all of his symptoms? How would you manage this patient?

**CASE #3**

You are called about a patient with acute respiratory distress and excruciating chest pain. It seemed to have started suddenly according to the nurse, but the patient is too breathless to talk. You read your signout: “24 yo M with ESRD, came in because his dialysis graft stopped functioning. Going to receive dialysis tomorrow.”

What do you think is going on?

What will you do now?

How will you assess further?

# **CASE #4**

A health young woman just had her first baby. She has called the nurse because she is having dizziness and palpitations.

What might be going on?

What one test do you need at this point?

Based on ECG # 4, what would your next few management steps be?

# **COMMON CALLS: ALTERED MENTAL STATUS**

### STUDENT COPY

**CASE # 1:**

You are called about a 74 year old woman with diabetes who was admitted one day ago with pneumonia and is now confused. On your check out sheet, the patient is listed as stable and improving, with plans to go home soon.

What questions would you like to ask the nurse?

As you are walking to see the patient, what is your differential for altered mental status?

What would you do now?

**CASE# 2:**

Mr. Smith is a 54 year old man brought to the hospital after he fell from a bridge and sustained a hip fracture. He underwent repair of the fracture, and now the nurse calls you three days into his hospital stay because he is mumbling and seeing things in the room that are not there.

What information do you need next, and what is your differential?

What additional history would you like?

What do you think is going on, and what would you do for the patient?

# **CASE #3**

The nurse calls you about Mrs. H, an 80 year old woman with a history of atrial fibrillation on coumadin who was admitted for chest pain. You are called overnight because she is found on the floor, confused.

# You go to see her right away. What is your differential?

Which study would you do?

What additional steps would you take?

When you look at the medication list you see that this patient received zolpidem 10 mg earlier that evening for sleep. What should you suggest as a first line treatment for sleep?

**Frequent Calls Student Guide**

**Fever**

**Case**

*You are called overnight about a 44 year-old woman with leukemia who has been in the hospital for 2 weeks for induction chemotherapy, who has a temperature of 101 degrees Fahrenheit.*

1. **What information do you need?**
2. **What should you do next?**
3. **What if the patient did not have leukemia and was admitted with chest pain?**

**Hypertension**

**Case 1**

*A 53-year-old female with end stage renal disease on intermittent emergent hemodialysis was admitted earlier in the day for signs of volume overload, typical of pre dialysis symptoms. She received one session of hemodialysis (removal of 2L) with plan for another session of dialysis in the morning. Overnight, the nurse calls you for elevated blood pressure to 168/90.*

**What would you ask the nurse?**

**What should you do next?**

**How should you treat the patient’s blood pressure?**

**Case 2**

*A 48-yo-male with HTN ran out of all his home meds about 2 weeks prior and presented to the EC earlier in the day with complaints of chest discomfort. His blood pressure at the time was 220/110. EKG did not show any signs of ischemic changes. He was given medication which improved his blood pressure and the chest discomfort resolved. Overnight, the nurse calls you to let you know that the patient’s blood pressure is now 190/100.*

**What would you ask the nurse?**

**What should you do next?**

**How should you treat this patient?**

**Pain**

**Case**

*A 33 year-old female is admitted for an asthma exacerbation. She has improved throughout her hospital course and the plan is for discharge tomorrow. The nurse calls you to report the patient is suffering from a headache and is requesting an order for a pain medication.*

**What do you do next?**

**What medications (if any) would you recommend?**

**Hyperglycemia**

**Case**

*At 9 pm, the nurse calls you about a 54 year old man with diabetes with a glucose of 360. From your check out sheet, you see that he was admitted today with chest pain.*

**What do you do next?**

**How would you manage this?**
