# Supplementary material for: Preparing for Medical Internship: A Case-Based Strategy to Teach Management of Common Overnight Calls to Students
Source: MedEdPORTAL. 2020 Sep 23;16:10966. doi: 10.15766/mep_2374-8265.10966 (PMC7511063; doi:10.15766/mep_2374-8265.10966)
Supplement: Supplementary file 1 — Facilitator Guides.docxHandout - Student Cases.docxRelevant Images (ECGs, Head CT).docxHandout - Student Tips.docxStudent Evaluation of Module.docx [file mep_2374-8265.10966-s001.zip › D. Handout - Student Tips.docx]

**Cross Coverage Tips for Common Calls & Orders During Internship**

- **Document your decisions / actions with a brief note (from one line to longer, depending)**
- **Never hesitate to call an upper level if uncertain or concerned about patient**
- **Always review current med list thoroughly before prescribing new meds**
- **Deprescribing may be as effective as prescribing. If (1) delirium, (2) urinary retention, or (3) constipation, look for (a) anticholinergics, (b) benzos, and (c) opioids and consider if they can be removed or reduced**
- **Always ask why, rather than just treating symptomatically / reflexively**
- **This document may guide you with a few first reflexes, but most calls warrant more nuanced consideration**
- **Clinicalproblemsolving.com (CPS below) has brief, high quality schema (approach-to) videos**

1. New/unexplained fever: focused hx/PE (lungs, bladder/abdomen, skin/lines), consider CXR, UA/UCx, BCx
   1. Acetaminophen (APAP) 1g PO. If concern for sepsis (e.g. + qSOFA) start empiric antibiotics
2. Pain: always consider etiology; if unknown or pain severe, see patient.
   1. APAP 1g PO safest, usually worth giving (alone or in addition to others). 2g/day safe in cirrhosis.
   2. Avoid NSAIDs (ibu, ketorolac) if AKI/CKD/HF/CAD/cirrhosis. Avoid tramadol in all.
   3. Severe pain – hydrocodone 5mg PO ~ oxycodone 2.5-5mg PO → morphine 2-4mg IV
3. Insomnia: sleep hygiene, treat pain (APAP 1g also has sleepy effect). Melatonin very safe. Trazodone 25mg next line. Avoid zolpidem, eszopiclone, benzos, esp if elderly or high risk delirium. Avoid diphenhydramine.
4. Itching: Review PO and IV meds, transfusions. Consider steroids cream if focal. Systemic: Diphenhydramine 25-50 mg po or Hydroxyzine 25-100mg po (anticholinergic – if elderly/risk, try high dose fexofenadine instead)
5. AMS: See the patient. Check vitals, glucose, meds, exam incl. neuro (if focal neuro -> CT head, stroke alert).
   1. Consider MIST: Metabolic (Na, Ca, Glc, ↑CO2, ↓O2, NH3), Infection, Stroke, Toxin (meds: anti-cholinergic, opiates, benzos, withdrawal). View CPS video.
6. Delirium: Redirect pt, sitter or family to bedside. Consider quetiapine 25mg po, olanzapine 2.5-5mg or haldol 0.5-2mg IM/IV; avoid lorazepam (unless alcohol withdrawal) or diphenhydramine – these worsen delirium
7. Falls: See patient, inquire re: head trauma. If yes or if abnormal neuro exam, stat CT head. Write note.
8. Seizure: call neurology; lorazepam 2mg IV if >5 minutes; can repeat q2min. Check glucose, lytes +/- CT
9. EtOH Withdrawal: lorazepam vs diazepam PRN per CIWA protocol
10. Chest Pain: Discuss with upper level. Consider ACS, PE, PNA, pneumothorax, Ao dissection, esoph perf. Stat EKG (compare to prior), see patient for focused hx/PE. Check trop now + in 2-6 hours. Consider CXR.
    1. If concern for ACS; give 0.4mg NTG SL, ASA 325, atorva 80, +/- LMWH / UFH.
11. Tachycardia: check ECG to discern sinus (ID and rx underlying case) vs. arrhythmia (rate control vs. convert)
    1. Sinus tach: volume ↓, bleed, sepsis, pain, MI, PE, HF, pericarditis, stroke. Do not slow with BBL.
    2. AF/AFL/A-tach/MAT w RVR: if BP ☺, metop 5mg IV or dilt 10-20 IV → follow with PO. If BP ↓, amio
    3. AVNRT/AVRT: modified Valsalva → adenosine 6mg → 12mg with continuous EKG
    4. Unstable tachyarrhythmia → RRT / ACLS / cardioversion
12. ↑BP: Check trend, evaluate for symptoms (CP, pulm edema, neuro), treat pain/nausea/anxiety/withdrawal
    1. If asx and <220/110 → rest, reassessment, restart home med or give PO dose early
    2. If HTN emergency (end-organ damage): may need IV labetalol or hydral. Alert UL, consider ICU.
13. ↓BP: See patient. Consider causes and effects. Call UL/RRT. Check if ↓baselines (small ppl, advanced cirrhosis).
    1. Bolus LR is first step (unless cardiogenic → CCU), but etiology must be found/treated
    2. Warm shins/forearms → sepsis/SIRS, anaphylaxis, adrenal insuff, neurogenic, vasodilators
    3. Cold shins/forearms + flat JVP → hypovolemic (bleed, diuresis)
    4. Cold shins/forearms + elevated JVP → cardiogenic vs obstructive (PE, tamponade, tension PTX).
14. Low UOP: Rule out urinary retention/obstruction (including clogged Foley).
    1. If context/exam suggest volume depletion/sepsis, 1L bolus. If volume overloaded, IV furosemide.
15. Hyperglycemia: Give short-acting insulin per sliding scale even if NPO. Recheck in 1h. Consider eval for DKA.
16. Hypoglycemia: Small juice first line. ½ amp D50 if very symptomatic or profound. Eval etiology, adjust insulin
17. Nausea/Vomiting: Consider etiology. Ondansetron 4mg IV q8, promethazine 12.5-25mg, prochlorperazine
18. GERD/Indigestion: Consider etiology incl atypical angina. Maalox, famotidine/ranitidine, omeprazole
19. Constipation: PO senna, bisacodyl, or miralax → bisacodyl suppository, lactulose, or mag citrate → enema (soap suds or tap, avoid fleets. Do not use docusate, good evidence it’s placebo.
20. Diarrhea: Check C. diff if sufficient pre-test. If known cause, not severe, not overflow: consider loperamide
21. GIB: See patient, visualize stool if possible, check VS and hgb stat and trend closely. Ensure 2 x 18G IVs. Start IVF. T&C blood, transfuse if large bleed. PPI. Consider coagulopathy/meds, reverse. Alert UL, GI +/- ICU.
22. Hgb drop: See patient, check vitals. If unexpected/mysterious, do rectal, recheck hgb. Ensure access, T&C.
    1. Transfuse if hgb < 7 or if large bleed with hemodynamic instability. No need to pre-med if no prior rx.
    2. If any concern for transfusion rx, stop immediately, alert blood bank to initiate dx/rx protocol
    3. If volume overloaded, consider administering blood with furosemide
23. Hyperkalemia: Check EKG. Call lab to ensure not hemolyzed, recheck if needed. “C-BIG-K”
    1. If ECG changes (TW↑, PR↔, PW↓,QRS↔), give 1gm CaGluc IV, repeat ECG, repeat Ca if need
    2. Push K into cells: D50 amp + insulin 10U reg IV (watch glu close), +/- IV bicarb if acidemic +/- albuterol
    3. Eliminate K from body: kayexalate 30gm po, furosemide IV if can tolerate, stat dialysis if oligoanuric
24. Hypokalemia: give 10mEq per 0.1 increase desired (unless CKD – reduce by ½). PO preferred. If severe, IV+PO
25. Hypomagnesemia: 2gm Mg Sulfate IV
26. Hypocalcemia: Correct # for albumin. Generally no need to give unless symptomatic (spasm, +Chvostek, etc)
27. Cough: Tessalon 100mg po tid; Guaifenesin 200-400mg po q4h; codeine if severe and not high risk.
28. Dyspnea or hypoxemia: see patient. ECG, CXR +/- ABG or VBG if suspect CO2 retention. Alert UL.
    1. O2 only for hypoxemia, titrate to 92-95% (cannula → ventimask → NRB)
    2. NIPPV (bipap) for severe COPD or HF exac, neutropenic PNA (avoid if altered or vomiting)
    3. If wheezing, consider nebs, but may also be from pulm edema
29. Patient wants to leave: Review team’s note. See patient, explore reasons, fix triggers if able. Discuss benefits of staying. Most have capacity to go unless severe dementia / delirium. Document in note.
30. Family wants update: Ask RN by when family will leave; relay time at which you can be at bedside. Read team’s note, show up, identify self as covering MD, discuss questions. Pass off other questions to AM team.
31. Telemetry is expired: Discontinue tele unless arrhythmia, HD instability, severe electrolyte issue.
32. Patient declining medications: Ask RN which meds and why pt declines. Discuss w pt if important meds, consider alternative agent. Ultimately, patient has right to decline.
