# Supplementary material for: Preparing for Medical Internship: A Case-Based Strategy to Teach Management of Common Overnight Calls to Students
Source: MedEdPORTAL. 2020 Sep 23;16:10966. doi: 10.15766/mep_2374-8265.10966 (PMC7511063; doi:10.15766/mep_2374-8265.10966)
Supplement: Supplementary file 1 — Facilitator Guides.docxHandout - Student Cases.docxRelevant Images (ECGs, Head CT).docxHandout - Student Tips.docxStudent Evaluation of Module.docx [file mep_2374-8265.10966-s001.zip › E. Student Evaluation of Module.docx]

#### STUDENT EVALUATION OF COMMON OVERNIGHT CALLS

#### The following modules helped prepare me to cross cover as an intern:

#### AMS 1 2 3 4 5

#### Strongly Disagree Strongly Agree

#### Chest Pain: 1 2 3 4 5

#### Strongly Disagree Strongly Agree

#### Frequent Calls: 1 2 3 4 5

#### Strongly Disagree Strongly Agree

#### After this session I feel more prepared for intern year:

#### 1 2 3 4 5

#### Strongly Disagree Strongly Agree

#### Format: 1 2 3 4 5

#### Ineffective Highly Effective

#### Comments to help us improve this session: ________________________________________________________________________________________________________________________________________________________________________________________________________________________________________________________________________________________________________________________________________________________________
